# Variation, coordination, and trade-offs between needle structures and photosynthetic-related traits across five *Picea* species: consequences on plant growth

**DOI:** 10.1186/s12870-022-03593-x

**Published:** 2022-05-17

**Authors:** Junchen Wang, Fangqun Ouyang, Sanping An, Lifang Wang, Na Xu, Jianwei Ma, Junhui Wang, Hanguo Zhang, Lisheng Kong

**Affiliations:** 1 State Key Laboratory of Tree Genetics and Breeding, Key Laboratory of Tree Breeding and Cultivation of State Forestry Administration, Research Institute of Forestry, Chinese Academy of Forestry, Beijing, 100091 People’s Republic of China; 2 Beijing Floriculture Engineering Technology Research Centre, Beijing Laboratory of Urban and Rural Ecological Environment, Beijing Botanical Garden, Beijing, 100093 China; 3 Research Institute of Forestry of Xiaolong Mountain, Gansu Provincial Key Laboratory of Secondary Forest Cultivation, Tianshui, 741022 People’s Republic of China; 4 State Key Laboratory of Tree Genetics and Breeding, Northeast Forestry University, Harbin, 150040 People’s Republic of China; 5 Department of Biology, Centre for Forest Biology, University of Victoria, Victoria, BC V8W 2Y2 Canada

**Keywords:** Needle structures, Biochemical parameters, Photosynthetic capacity, Photosynthetic N allocation, PNUE, Tree growth

## Abstract

**Background:**

*Picea* species are distributed and planted world-wide due to their great ecological and economic values. It has been reported that *Picea* species vary widely in growth traits in a given environment, which reflects genetic and phenotypic differences among species. However, key physiological processes underlying tree growth and the influencing factors on them are still unknown.

**Results:**

Here, we examined needle structures, needle chemical components, physiological characteristics and growth traits across five *Picea* species in a common garden in Tianshui, Gansu province in China: *Picea glauca*, *P. mariana*, *P. likiangensis*, *P. koraiensis*, and *P. crassifolia*, among which *P. glauca* and *P. mariana* were introduced from North America, *P. likiangensis* was from Lijiang, Yunan province in China, *P. koraiensis* was from Yichun, Heilongjiang province in China, and *P. crassifolia* was native to the experimental site. It was found that nearly all traits varied significantly among species. Tissue-level anatomical characteristics and leaf mass per area (LMA) were affected by needle size, but the variations of them were not associated with the variations in photosynthetic and biochemical capacity among species. Variations in area-based maximum photosynthesis (P_nmax_) were affected by stomatal conductance (g_s_), mesophyll conductance (g_m_) and biochemical parameters including maximum carboxylation rate (V_cmax_), and maximum electron transport rate (J_max_). The fraction of N allocated to different photosynthetic apparatus displayed contrasting values among species, which contributed to the species variations in photosynthetic nitrogen use efficiency (PNUE) and P_nmax_. Additionally, all growth traits were positively correlated with P_nmax_ and PNUE.

**Conclusion:**

Needle structures are less important than needle biochemical parameters in determining the variations in photosynthetic capacity across the five *Picea* species. P_nmax_ and PNUE are closedly associated with the fraction of N allocated to photosynthetic apparatus (P_photo_) compared with leaf N content per area (N_area_). The tremendous growth differences among the five *Picea* species were substantially related to the interspecies variation in P_nmax_ and PNUE.

**Supplementary Information:**

The online version contains supplementary material available at 10.1186/s12870-022-03593-x.

## Introduction

*Picea* A. Dietrich (spruce) is the third largest genus in *Pinaceae* and consists of approximately 40 species mainly distributed in temperate regions and middle and high mountainous area of northern subtropical hemisphere [1, 2]. The largest center of distribution and differentiation of *Picea* is in Asia, with the largest number of species occurring in China [1], including 16 species and 9 variants. It plays a substantial role in the boreal and sub-alpine forests in the northern hemisphere and features high economical value due to its high-quality timber [3]. Spruce introductions have started in China since the 1980s, mainly including early evaluation and species or provenance selection [4–6]. Interestingly, in these trials, some exotic (non-native) species exhibit strong adaptability and good growth performance in some regions of China such as Gansu and Jilin province [3, 5–7]. Similar trends were also revealed in the report of Holst [8], in which the growth of the introduced species, *P. abies*, could surpass native spruce species in North America. In the light of the important role *Picea* species play in ecology and economy, an enhanced knowledge of the physiological mechanisms underlying the interspecific growth differences may increase our ability to develop proper management plans for spruce introduction and breeding.

Plant survival and development are based on physiological processes and many of these occur within leaves, where the ability to maintain hydraulics, conduct photosynthesis, regulate temperature, gather and harvest light etc. are dependent on leaf structures [9, 10]. It is widely accepted that leaf anatomy and mesophyll properties can affect carbon assimilation and leaf thickness are strongly correlated with leaf area-based photosynthesis [11–13]. As the key organs of conifers [14], many studies have shown that needle structures may vary tremendously among and within species e.g.,[7, 9, 15], and these variations may have important implications for shaping plant functioning [16, 17]. Considering the multi-faceted roles needles play, it is expected that needle structures should strongly affect the physiological functions at the leaf level, among which photosynthesis is an essential process. Convincing evidence has suggested that improving photosynthesis can promote plant growth although it is controversial whether plant growth is mainly limited by photosynthesis, i.e., the ‘source’, or by demand of carbohydrates from new vegetative growth or the developing seeds and fruits, i.e., the ‘sink’ [18, 19]. The most accepted view at present is that photosynthesis is a major driver of plant growth and productivity as it is the origin of carbon skeletons for plants to build up all their structures for both maintenance and growth [19], thus making it one of the most studied processes in plant physiology. Amongst leaf structural aspects, leaf mass per unit area (LMA) has been considered a vital functional trait due to its negative effects on the mass-based photosynthesis [20, 21], but how LMA affects area-based photosynthesis (P_n_) remains less clear when considered globally [22]. Moreover, although LMA has been demonstrated to be negatively correlated with mesophyll conductance (g_m_) [23, 24], an essential limiting factor for P_n_, this is not always true because LMA is a highly integrated trait. Different anatomical changes may lead to the uncertain relationship between LMA and g_m_ [24, 25]. Hence, whether leaf structures impose strong constraints on leaf functions remains empirical. From a functional perspective, needle internal structures should be directly related to physiological processes [26, 27] and may have important implications for foliage potential photosynthesis [28–30]. However, little research has been conducted on the correlations between needle traits and photosynthetic characteristics at interspecies level in genus *Picea*, which may be essential for understanding the variation in growth rates and adaptive capacities across species.

Nitrogen (N) is one of the most important mineral nutrients that limit the growth of plants in many parts of the world [31]. The economics of N use in photosynthesis has become a research hotspot in the past several decades and has continued to this day [32, 33]. As much as 75% of leaf N is invested into photosynthetic apparatus, with an average 20% invested in ribulose 1,5-bisphosphate carboxylase (Rubisco) [31, 34–37]. Strong positive correlation relationship between light-saturated net CO_2_ assimilation rate (P_nmax_) and leaf nitrogen content per unit area (N_area_) observed in many species previously [38, 39] indicate that leaf photosynthesis may be controlled by the supply and demand of leaf N content to a large extent. Photosynthetic-nitrogen use efficiency (PNUE), defined as the ratio of P_nmax_ to N_area_, has been regarded as an important leaf trait for characterizing plant leaf economics, physiology, and survival strategy [40]. Species with high PNUE tend to have higher growth rates [40] and higher competitive ability in natural ecosystems [41]. Additionally, interspecific variation of P_nmax_ and PNUE is related to N allocation in the photosynthetic apparatus (P_photo_, composed of leaf N allocated to Rubisco (P_C_), bioenergetics (P_B_) and light-harvesting components (P_L_)) [42, 43]. Although genus *Picea* has been transplanted worldwide due to its huge economic and ecological values [7], to our knowledge, little information is available on how *Picea* species differ in photosynthetic N allocation, P_nmax_ and PNUE, which may be directly related to the growth discrepancies among species.

In the present study, we employed five spruce species as our tested materials, out of which two were from North America (*P. glauca* and *P. mariana*), one from Heilongjiang province in China (*P. koraiensis*), one from Yunnan province in China (*P. likiangensis*), and one native to our experimental site (*P. crassifolia*), and cultivated them in the National Germplasm Repository Germplasm Resources Preservation of spruce in Xiaolong Mountain of Gansu province of China. Our previous research has revealed the significant growth differences among these species [3, 7]. We hypothesize that the five *Picea* species vary widely in needle structures, biochemical parameters and photosynthetic-related traits and there is co-variation among these traits. In addition, the interspecies growth differences are closely related to the variation in photosynthetic capacity and PNUE among the five *Picea* species. The objectives of the present study were to (1) reveal the variation in needle structures, photosynthetic characteristics, photosynthetic N allocation and growth traits; (2) clarify the relationship among needle structures, biochemical parameters and photosynthetic-related traits; and (3) evaluate the consequences of these relationship on plant growth.

## Results

### Variation in needle structural traits among species and their correlations

The studied species displayed contrasting values of key needle structural traits. All traits except ETF were all significantly different among species (Table 1). According to Duncan’s multiple range test, *P. likiangensis* had the largest NL (11.46 mm), NW (0.96 mm), NSA (0.80 mm^2^), NSC (3.58 mm), MA (0.60 mm^2^) and ETA (0.14 mm^2^) while exhibited the smallest CCF (5.18%) and TMF (23.42%). *P. glauca* had the largest SD (60.93 No./ mm^2^), and ETA (0.04 mm^2^) but the smallest NW (0.78 mm), MF (65.89%), and ETT (0.03 mm). In contrast, *P. mariana* had the smallest NL (8.24 mm), NW (0.79 mm), NT (0.79 mm), NSA (0.38mm^2^), MA (0.26 mm^2^), and ETA (0.08mm^2^). The native species, *P. crassifolia*, had the largest LMA (0.027 g/cm^2^) but the smallest SD (32.57 No./mm^2^) among the five species.

**Table 1:** Needle structures for the five studied *Picea* species

Traits (Unit)	*P. glauca*	*P. mariana*	*P. likiangensis*	*P. koraiensis*	*P. crassifolia*	Significance (ANOVA)
NL (mm)	1.10 ± 0.10ab	0.82 ± 0.12c	1.15 ± 0.15a	0.98 ± 0.04b	1.02 ± 0.12b	**
NW (mm)	0.78 ± 0.08b	0.79 ± 0.10b	0.96 ± 0.06a	1.00 ± 0.09a	1.02 ± 0.08a	**
NT (mm)	0.91 ± 0.05c	0.79 ± 0.05d	1.03 ± 0.08ab	1.00 ± 0.02b	1.10 ± 0.07a	**
SD (No./mm^2^)	60.93 ± 5.67a	51.54 ± 0.63b	49.81 ± 2.48b	41.51 ± 1.95c	32.57 ± 2.32d	**
NSA (mm^2^)	0.51 ± 0.04c	0.38 ± 0.02d	0.80 ± 0.070a	0.61 ± 0.01b	0.64 ± 0.10b	**
MA (mm^2^)	0.34 ± 0.02c	0.26 ± 0.02d	0.60 ± 0.05a	0.43 ± 0.01b	0.46 ± 0.06b	**
ETA (mm^2^)	0.12 ± 0.03a	0.08 ± 0.01b	0.14 ± 0.02a	0.12 ± 0.02a	0.12 ± 0.03a	*
CCA (mm^2^)	0.040 ± 0.009bc	0.033 ± 0.004c	0.041 ± 0.005bc	0.044 ± 0.002b	0.056 ± 0.007a	**
TMA (mm^2^)	0.16 ± 0.04a	0.11 ± 0.00b	0.19 ± 0.02a	0.17 ± 0.02a	0.17 ± 0.03a	*
MF (%)	65.89 ± 5.62c	67.75 ± 1.75bc	75.38 ± 2.10a	70.47 ± 2.41bc	71.59 ± 1.12ab	**
ETF (%)	23.97 ± 5.41	20.34 ± 1.62	17.84 ± 1.91	20.27 ± 2.56	17.61 ± 2.02	NS
CCF (%)	7.78 ± 1.20ab	8.73 ± 1.26a	5.18 ± 0.36c	7.22 ± 0.47b	8.74 ± 0.81a	**
TMF (%)	31.75 ± 5.62a	29.06 ± 1.63ab	23.42 ± 1.32c	27.49 ± 2.57bc	26.35 ± 1.24bc	*
LMA (g/m^2^)	180.34 ± 18.65c	167.93 ± 11.99c	158.10 ± 4.42c	229.67 ± 33.74b	268.50 ± 29.16a	**

*NL* needle length, *NW* needle width, *NT* needle thickness, *SD* stomatal density, *NSA* needle section area, *ETA* and *ETF* epidermis tissue area and fraction, *MA* and *MF* mesophyll area and fraction, *CCA* and *CCF* central cylinder area and fraction, *TMA* and *TMF* total mechanical tissue area and fraction. Data are mean ± SE. Different letters indicate significant differences among species (*P* < 0.05). *, statistical significance at *P* < 0.05; **, statistical significance at *P* < 0.01

**Fig. 1:**
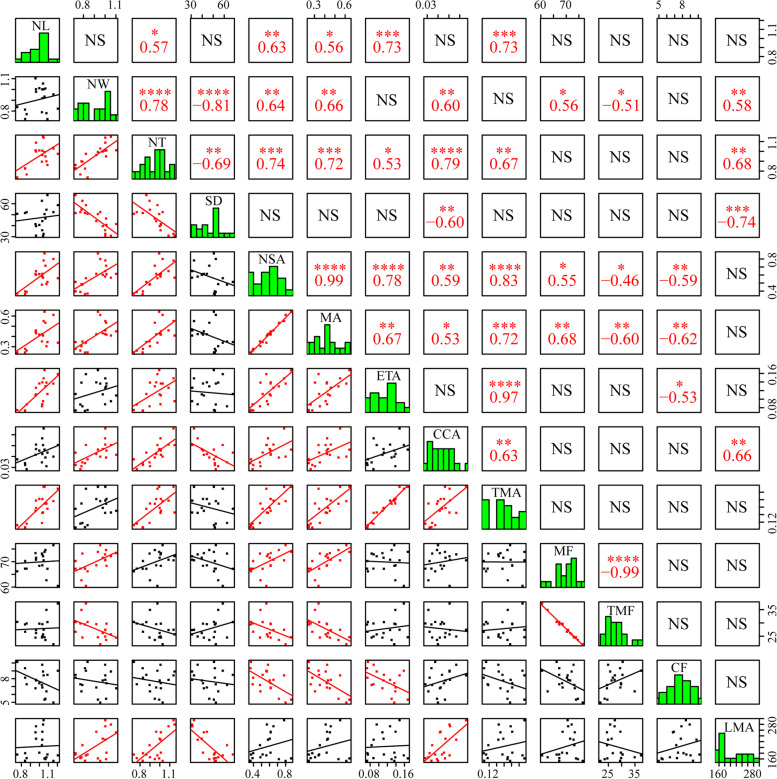
Correlation coefficients between needle structures. Histograms show trait distributions and correlations. NL, needle length; NW, needle width; NT, needle thickness; SD, stomatal density; NSA, needle section area; ETA, epidermis tissue fraction; MA and MF, mesophyll area and fraction; CCA and CCF, central cylinder area and fraction; TMA and TMF, total mechanical tissue area and fraction. *, statistical significance at *P* < 0.05; **, statistical significance at *P* < 0.01; ***, statistical significance at *P* < 0.001, ****, statistical significance at *P* < 0.0001

Pearson correlation analysis were conducted based on needle structural traits with significant difference among species. NT, NSA, MA, TMA and ETA were significantly positively correlated with NL. NW and NT both showed significantly positive correlations with NSA, MA, CCA and LMA while showed negative correlations with SD. In addition, LMA also showed strong positive correlations with CCA. NSA, MA and CCA were positively correlated with each other while NSA, MA and ETA were negatively correlated with CF (Fig. 1).

### Needle chemical traits

**Fig. 2:**
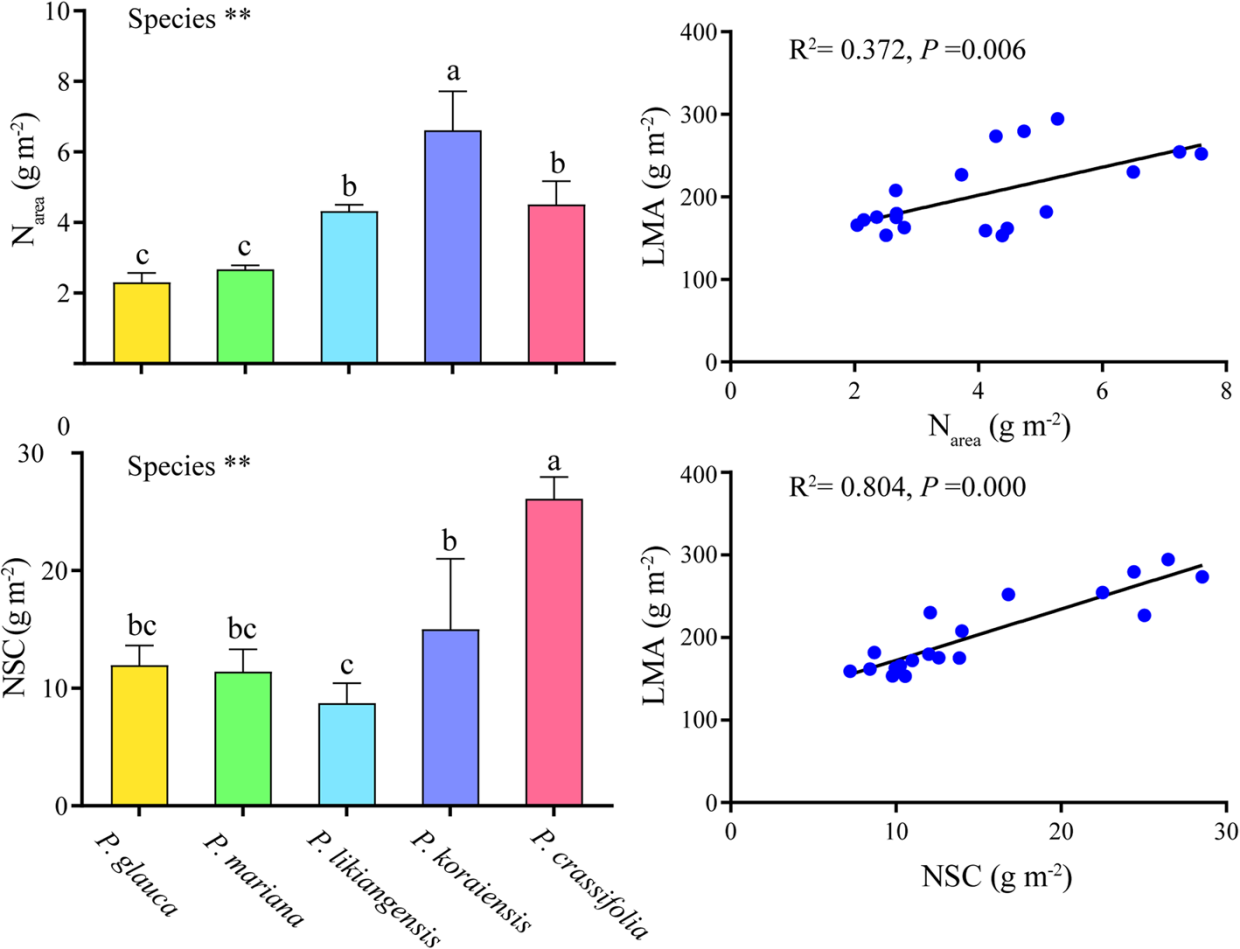
Comparison of N_area_ (leaf N content per area) and NSC (non-structural carbohydrate content) among species and linear relationship between LMA and N_area_ as well as NSC. N_area_, leaf N content per area; NSC, non-structural carbohydrate content; LMA, leaf mass per area. Different letters indicate significant differences among species (*P* < 0.05). *, statistical significance at *P* < 0.05; **, statistical significance at *P* < 0.01

The five tested species showed significant differences in needle chemical traits including N_area_ (g m^− 2^) and NSC (g m^− 2^). *P. koraiensis* exhibited the largest N_area_ (6.61 g m^− 2^) while *P. glauca* and *P. mariana* exhibited the lowest (2.30 and 2.67 g m^− 2^, respectivly). *P. crassifolia* had the largest NSC (26.11 g m^− 2^) while *P. likiangensis* had the lowest (8.73 g m^− 2^). In addition, NSC showed a tight positive linear relationship with LMA (*R* ^2^ = 0.804, *P* = 0.000) whereas a weak correlation was detected between N_area_ and LMA (*R* ^2^ = 0.372, *P* = 0.006) (Fig. 2).

### Photosynthetic and biochemical parameters

**Fig. 3:**
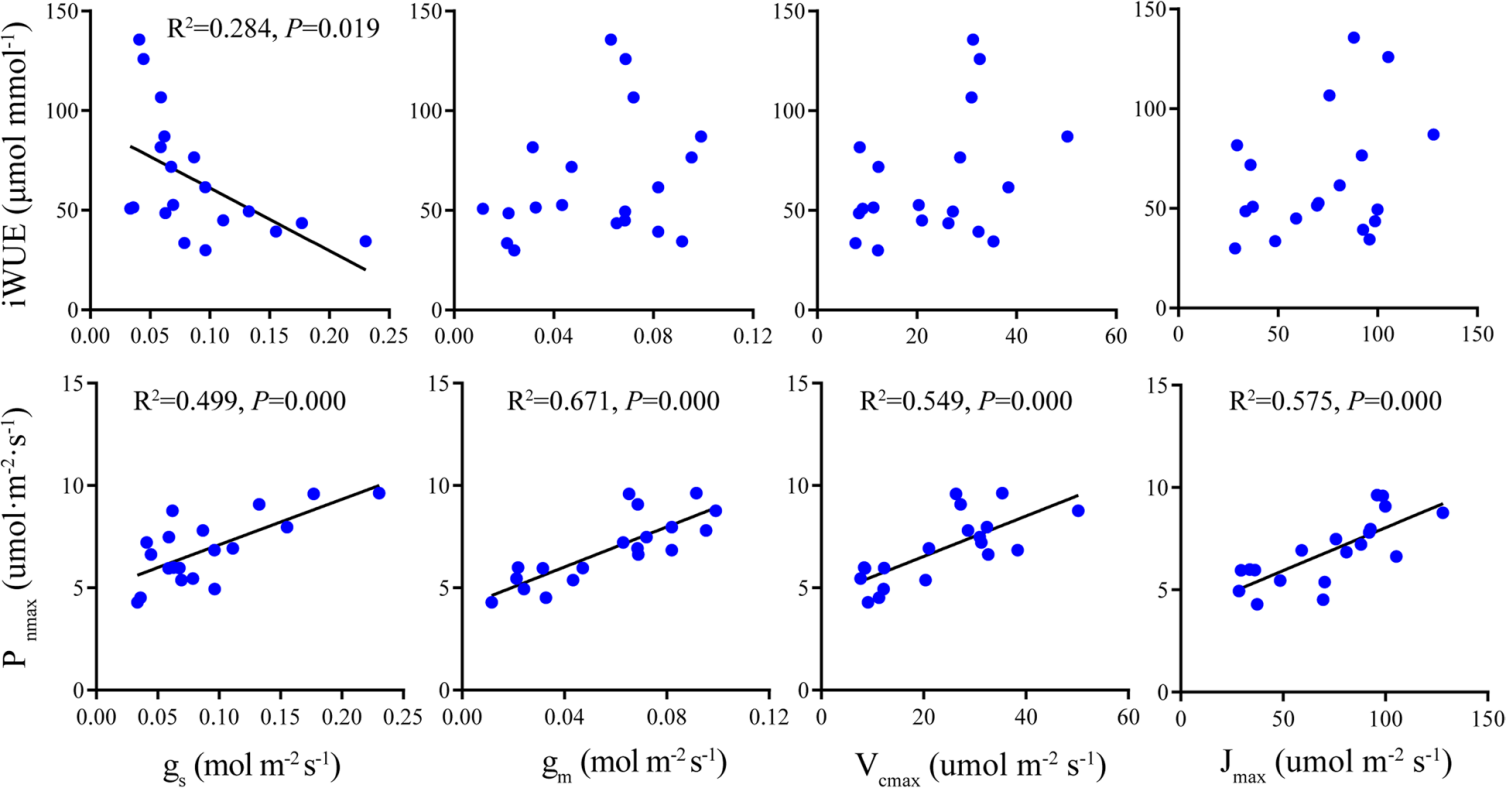
Relationships between biochemical parameters and photosynthetic capacity across species. P_nmax_, area-based maximum net photosynthesis; g_s_, stomatal conductance; iWUE, intrinsic water use efficiency; g_m_, mesophyll conductance to CO_2_; V_cmax_ and J_max_, maximum carboxylation and maximum electron transport rate, respectively. Points represent trait values for each individual of each species. Explained variance (R^2^) and *P* values are shown. Solid lines in each panel represents significant linear regression

**Fig. 4:**
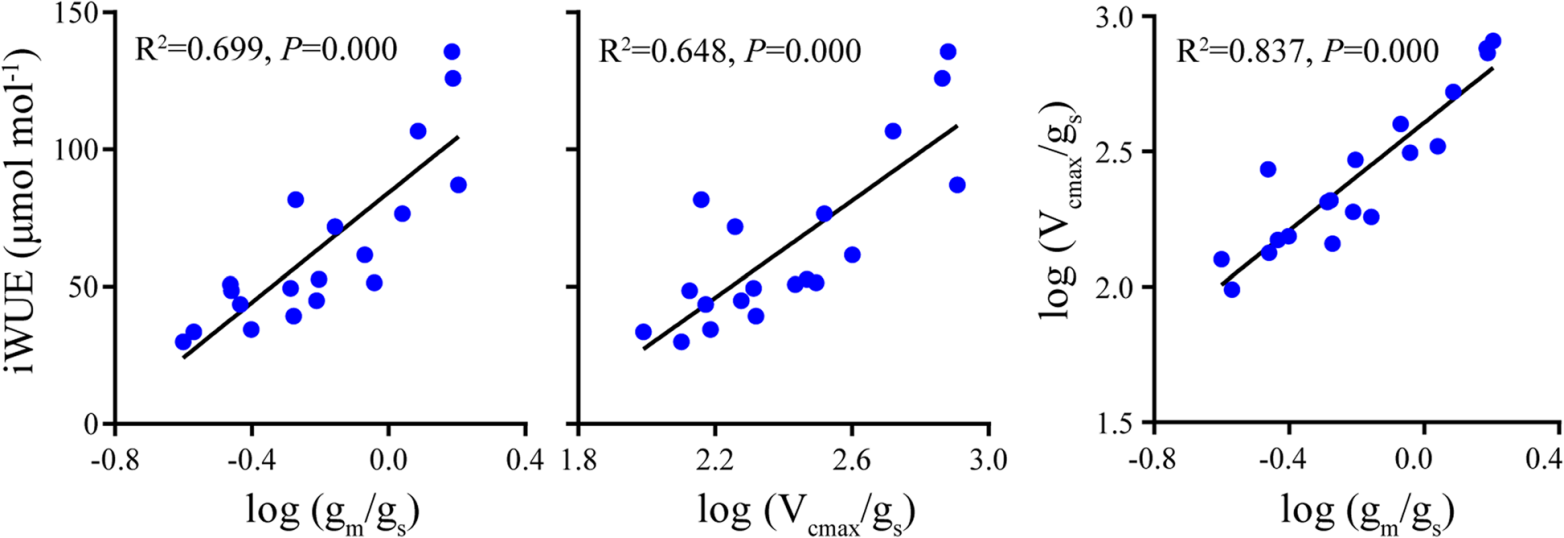
Relationships between log (g_m_/g_s_) as well as log (V_cmax_/g_s_) and iWUE and the relationship between log (g_m_/g_s_) and log (V_cmax_/g_s_). g_s_, stomatal conductance; g_m_, mesophyll conductance to CO_2_; V_cmax_ and J_max_, maximum carboxylation and maximum electron transport rate, respectively. Points represent trait values for each individual of each species. Explained variance (R^2^) and *P* values are shown. Solid lines in each panel represents significant linear regression

*Picea* species vary considerably in photosynthetic (P_nmax_ and iWUE) and biochemical parameters (g_s_, g_m_, V_cmax_ and J_max_) (Table 2). Specifically, *P. glauca* showed the largest P_nmax_ (9.27 umol m^− 2^ s^− 1^) while *P. mariana* and *P. crassifolia* showed the lowest (5.57 and 5.07 umol m^− 2^ s^− 1^, respectively). *P. likiangensis* exhibited the largest iWUE (122.78 μmol mmol^− 1^) while iWUE of the remaining four species were not statistically different. V_cmax_, J_max_ and g_m_ of *P. glauca*, *P. likiangensis* and *P. koraiensis* were significantly higher than those of *P. mariana* and *P. crassifolia* (Table 2). To summarize, *P. glauca*, *P. likiangensis*, and *P. koraiensis* exhibited greater photosynthetic capacities in the five tested species, that is, higher P_nmax_, V_cmax_, and J_max_ values. The correlation relationship between photosynthetic and biochemical parameters showed that increased iWUE was associated with lower gs while not correlated with g_m_, V_cmax_ and J_max_. In contrast, P_nmax_ was significantly positively correlated with g_s_, g_m_, V_cmax_ and J_max_ and the correlation between P_nmax_ and g_m_ (*R*^2^ = 0.671, *P* < 0.01) was stronger than that between P_nmax_ and g_s_, V_cmax_ as well as J_max_ (*R*^2^ = 0.499, *P* = 0.000; *R*^2^ = 0.549, *P* = 0.000; *R*^2^ = 0.575, *P* = 0.000) (Fig. 3). P_nmax_ showed no correlation relationship with iWUE. We used log values to evaluate the relationship between iWUE and g_m_/g_s_ as well as V_cmax_/g_s_ and the results showed that both were significantly positively correlated with iWUE (*R*^2^ = 0.699, *P* = 0.000; *R*^2^ = 0.648, *P* = 0.000) (Fig. 4).

**Table 2:** Photosynthetic characteristics for the five studied *Picea* species

Traits	*P.glauca*	*P. mariana*	*P. likiangensis*	*P. koraiensis*	*P.crassifolia*	significance (ANOVA)
P_nmax_ (umol m^− 2^ s^− 1^)	9.27 ± 0.42a	5.57 ± 0.50c	7.11 ± 0.43b	7.39 ± 0.58b	5.07 ± 0.80c	**
iWUE (μmol mol^−1^)	53.62 ± 23.11b	59.02 ± 22.79b	122.78 ± 14.75a	55.61 ± 16.88b	46.10 ± 8.43b	**
g_s_ (mol m^−2^ s^−1^)	0.150 ± 0.071a	0.073 ± 0.016b	0.048 ± 0.01b	0.112 ± 0.03ab	0.053 ± 0.022b	*
g_m_ (mol m^−2^ s^−1^)	0.081 ± 0.017a	0.037 ± 0.011b	0.068 ± 0.005a	0.082 ± 0.011a	0.022 ± 0.009b	**
V_cmax_ (umol m^−2^ s^−1^)	34.79 ± 11.05a	13.32 ± 5.02b	31.62 ± 0.9a	30.12 ± 7.26a	9.11 ± 1.55b	**
J_max_ (umol m^−2^ s^−1^)	105.69 ± 15.05a	41.11 ± 19.78b	89.74 ± 14.82a	81.20 ± 15.72a	47.29 ± 16.11b	**

P_nmax_, area-based maximum net photosynthesis; g_s_, stomatal conductance; iWUE, intrinsic water use efficiency; g_m_, mesophyll conductance to CO_2_; V_cmax_ and J_max_, maximum carboxylation and maximum electron transport rate, respectively. Data are mean ± SE. Different letters indicate significant differences among species (*P* < 0.05); *, statistical significance at *P* < 0.05; **, statistical significance at *P* < 0.01

### Photosynthetic N allocation and PNUE

The N allocation in photosynthetic apparatus exhibited different modes among the five species. In general, P_C_, P_B_, P_L_, P_photo_, P_non-photo_ and PNUE were all significantly different among species and P_C_ was larger than P_B_ and P_L_ in all species (Table 3). Among the five species, *P. glauca* had the largest P_L_, P_B_, P_C_ and P_photo_ (2.74, 3.71, 11.96 and 18.41%, respectively) while *P. crassifolia* had the lowest P_L_ and P_photo_ (0.74 and 3.23%, respectively). P_B_, P_L_, P_photo_ and P_non-photo_ of *P. mariana*, *P. likiangensis*, *P. koraiensis* were not significantly different statistically. *P. glauca* exhibited the largest PNUE (56.89 μmol mol^− 1^ s^− 1^) while *P. koraiensis* and *P. crassifolia* had the lowest among species (15.93 and 16.22 μmol mol^− 1^ s^− 1^, respectively) (Table 3).

**Table 3:** Photosynthetic N allocation and PNUE for the five studied *Picea* species

Traits	*P.glauca*	*P. mariana*	*P. likiangensis*	*P. koraiensis*	*P.crassifolia*	Significance (ANOVA)
P_C_ (%)	11.96 ± 4.71a	3.83 ± 1.41b	5.65 ± 0.4b	3.68 ± 1.51b	1.64 ± 0.14b	**
P_B_ (%)	3.71 ± 0.77a	1.23 ± 0.59b	1.66 ± 0.34b	1 ± 0.26b	0.85 ± 0.3b	**
P_L_ (%)	2.74 ± 0.6a	1.66 ± 0.61bc	2.16 ± 0.36ab	1.01 ± 0.1 cd	0.74 ± 0.17d	**
P_photo_ (%)	18.41 ± 5.93a	6.72 ± 1.92bc	9.47 ± 1.03b	5.69 ± 1.74bc	3.23 ± 0.35d	**
P_non-photo_ (%)	81.59 ± 5.93c	93.28 ± 1.92ab	90.53 ± 1.03b	94.31 ± 1.74ab	96.77 ± 0.35a	**
PNUE (μmol mol^−1^ s^−1^)	56.89 ± 6.69a	29.34 ± 3.73b	23.03 ± 0.74b	15.93 ± 2.38c	16.22 ± 4.52c	**

P_C_, P_B_, P_L_, P_photo_ and P_non-photo_, the fraction of leaf N allocated to Rubisco, bioenergetics, the light-harvesting components, photosynthetic apparatus and non-photosynthetic apparatus (%), respectively. PNUE, photosynthetic nitrogen use efficiency. P_photo_ = P_L_ + P_B_ + P_C_; P_non-photo_ = 1-P_photo_. Data are mean ± SE. Different letters indicate significant differences among species (*P* < 0.05); *, statistical significance at *P* < 0.05; **, statistical significance at *P* < 0.01

**Fig. 5:**
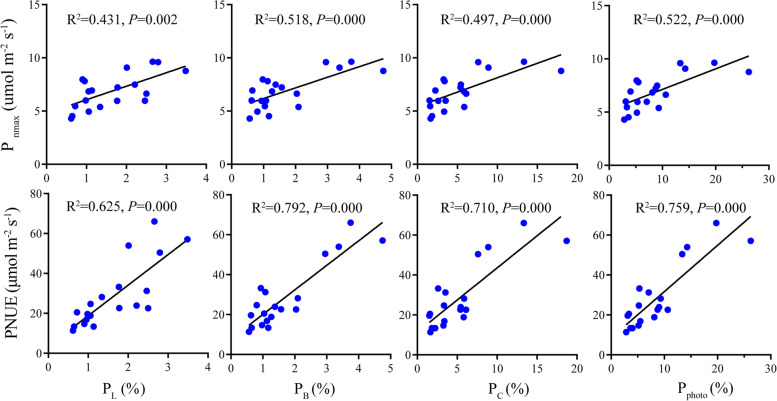
Relationships between the fraction of N allocated to different photosynthetic apparatus and P_nmax_ and PNUE. P_C_, P_B_, P_L_ and P_photo,_ the fraction of leaf N allocated to Rubisco, bioenergetics, the light-harvesting components and photosynthetic apparatus (%), respectively; P_nmax_, area-based maximum net photosynthesis; PNUE, photosynthetic nitrogen use efficiency. Points represent trait values for each individual of each species. Explained variance (R^2^) and *P* values are shown. Solid lines in each panel represents significant linear regression

The results of linear regression analysis showed that both P_nmax_ and PNUE were significantly positively correlated with P_L_, P_B_, P_C_ and P_photo_, but the correlation relationship between P_L_, P_B_, P_C_ as well as P_photo_ and PNUE (*R*^2^ = 0.625, *P* = 0.000; *R*^2^ = 0.792, *P* = 0.000; *R*^2^ = 0.710, *P* = 0.000; *R*^2^ = 0.759, *P* = 0.000) were stronger than that between P_L_, P_B_, P_C_ as well as P_photo_ and P_nmax_ (*R*^2^ = 0.431, *P* = 0.002; *R*^2^ = 0.518, *P* = 0.000; *R*^2^ = 0.497, *P* = 0.000; *R*^2^ = 0.522, *P* = 0.000) (Fig. 5).

#### Species differences in growth traits

**Fig. 6:**
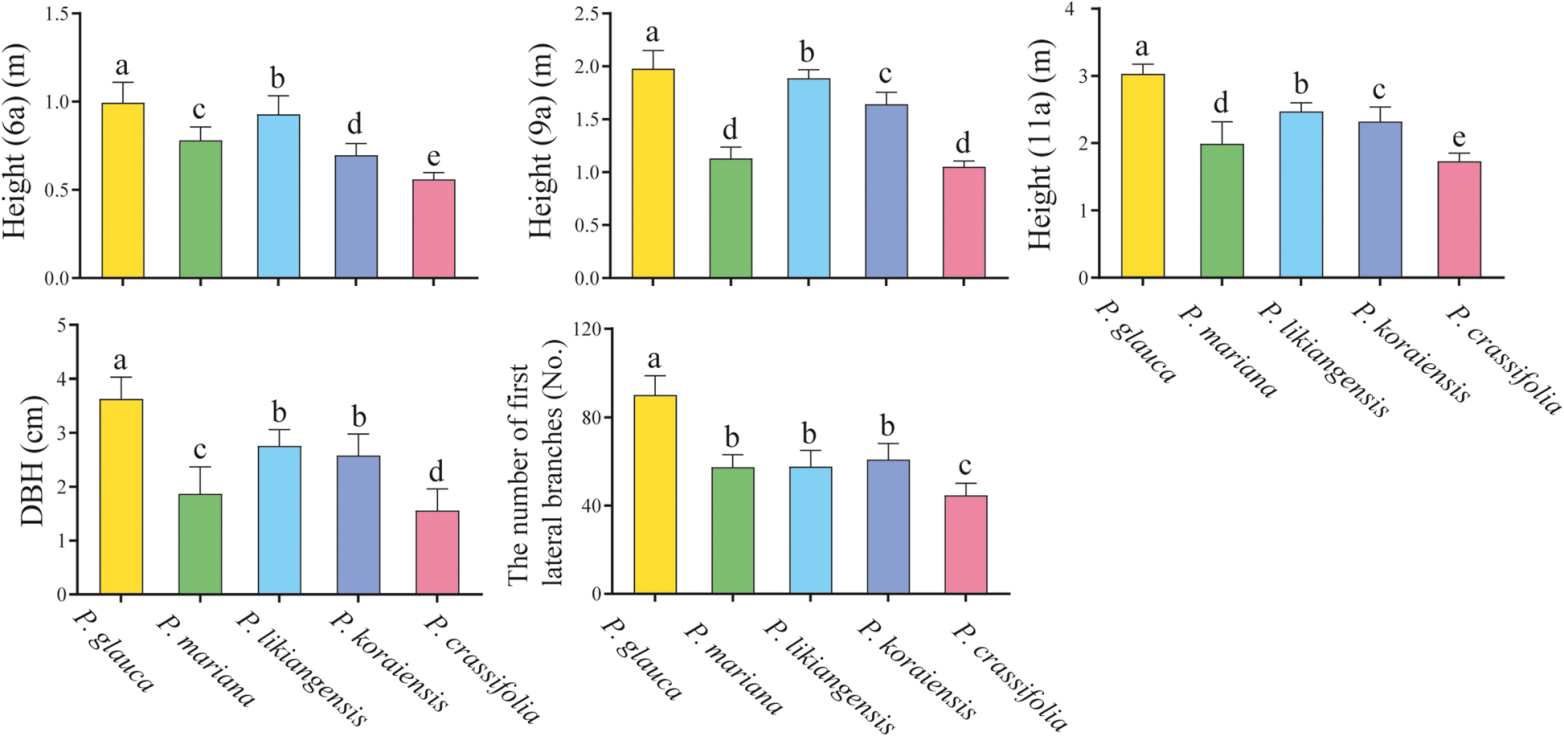
Comparison of growth traits among species. Height (6a), Height (9a) and Height (11a), tree height at age 6, 9, and 11, respectively; DBH, diameter at breast height. Different letters indicate significant differences among species (*P* < 0.05). *, statistical significance at *P* < 0.05; **, statistical significance at *P* < 0.01

**Fig. 7:**
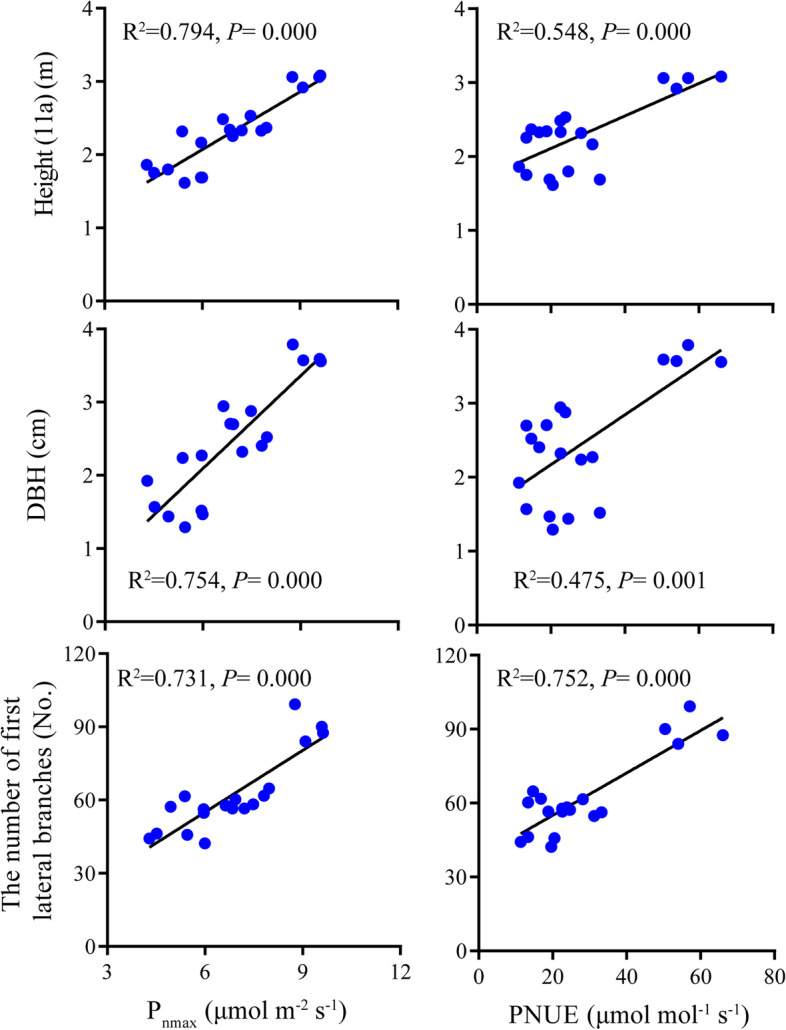
Relationships between growth traits and P_nmax_ as well as PNUE. Height (11a), tree height at age 11; DBH, diameter at breast height; P_nmax_, area-based maximum net photosynthesis; PNUE, photosynthetic nitrogen use efficiency. Points represent trait values for each individual of each species. Explained variance (R^2^) and *P* values are shown. Solid lines in each panel represents significant linear regression

The results of ANOVA showed that tree height at age 6, 9 and 11, DBH, and the number of first lateral branches were all significantly different among species, and tree height at age 11 was significantly different among blocks (Table 4). Moreover, the interactions of species and blocks were significant for height (11a) and DBH. *P. glauca* showed the largest tree height at age 6, 9 and 11, DBH and the number of first lateral branches among the five species while these traits were all the lowest for *P. crassifolia*. To summarize, at our experimental site, *P. glauca* exhibited the best growth performance among species, with *P. likiangensis* and *P. koraiensis* the middle and *P. mariana* as well as *P. crassifolia* the lowest (Fig. 6). Additionally, significantly positive linear relationship was observed between P_nmax_ as well as PNUE and growth traits. Thus, the growth discrepancies among the five studied species were primarily driven by P_nmax_ and PNUE. However, the correlation relationship between height (11a) as well as DBH and P_nmax_ (*R*^2^ = 0.794, *P* = 0.000; *R*^2^ = 0.754, *P* = 0.000) was stronger than that between the two growth traits and PNUE (*R*2 = 0.548, *P* = 0.000; *R*^2^ = 0.475, *P* = 0.001), whereas both P_nmax_ and PNUE were tightly correlated with the number of first lateral branches (*R*^2^ = 0.731, *P* = 0.000; *R*^2^ = 0.731, *P* = 0.000) (Fig. 7).

**Table 4:** ANOVA of growth traits for the five studied *Picea* species

Traits	Species		Block		Species× Block		Error
	MS	*F*	MS	*F*	MS	*F*	(MS)
Height (6a)	0.49	78.37**	0.03	0.01	0.13	0.01	0.01
Height (9a)	11.71	2.93**	0.04	0.01	0.12	0.01	0.01
Height (11a)	15.74	3.93**	0.33	0.11*	1.08	0.09*	0.03
DBH	10.48	82.63**	0.37	2.65	0.31	2.44*	0.13
The number of first lateral branches	18,194.20	4548.55**	187.50	62.5	662.00	55.17	46.28

Height (6a), Height (9a) and Height (11a), tree height at age 6, 9, and 11, respectively. DBH, diameter at breast height. *, statistical significance at *P* < 0.05; **, statistical significance at *P* < 0.01

## Discussion

Our results clearly showed the interspecies variation for needle structures, chemical components, photosynthetic capacities, biochemical parameters, photosynthetic N allocation and growth traits as well as the coordination or trade-offs between them. To our knowledge, this work represents the first relatively comprehensive study trying to figure out all these relationships together among multiple species within genus *Picea*. The variations in these characteristics and the correlations between them may make it possible for tree breeders to select the most suitable species for a given environment, for example, our experimental site with a temperate subhumid climate.

We assume that the significant differences observed in needle structural characteristics were mainly attributed to genetic factors as needle structures are relatively stable at species level [44]. In addition, the significant correlation relationship between some traits (Fig. 1) may indicate the constraints of needle structural design. Leaf size affects many functional traits and is related to plant productivity and adaptive capacity to changes in its environment [45], but this has been seldom studied in genus *Picea*. Considering the great differences of pine needle length among species, Wang et al. reported that NL was positively correlated with mechanical tissue fraction and area while negatively correlated with MA and MF in five southern pine species, suggesting that increasing needle stiffness in these species came at a cost of photosynthetic tissue (mesophyll) when NL increased [9], but this was on the premise that NSA did not increase with NL. Similar trade-offs based on findings on tissue fractions in three Mediterranean pines were also reported by Kuusk et al. [46]. Although MA, ETA and TMA were found to be positively correlated with NL in our results, but the variation of them along needle length across species was also significantly integrated by NW or NT, and CCA was only affected by NW and NT regardless of NL. Moreover, NW was also detected to be significantly correlated with relative indicators including MF and TMF. These results may indicate that, for the studied *Picea* species, variations in needle internal structures should be largely, at least partially, dependent on NW and NT despite the fact that NL do have a certain impact on some traits. Moreover, LMA was also found to be positively related to NW and NT. In other words, needle anatomical changes are related to morphological adjustments [9] and the resulting coordination and trade-offs between traits may be the consequence of the interaction of various indicators regarding needle size. Needles must be rigid and flexible enough to support their own weight as well as bend or twist without breaking when subjected to external forces [47, 48], which could be reflected by increasing TMA when NL increased in this study. However, *Picea* species show a much narrower range of NL (0.82–1.15 cm in this study) in *Pinaceae* comparing with genus *Pinus* (e.g., 7.51–34.59 cm in [9]), thus it’s unlikely for *Picea* species to invest obviously more to needle supporting tissues at the cost of photosynthetic tissue (mesophyll) when NL increase. Additionally, our results also showed that NL, NW and NT positively affected NSA unevenly while NSA simultaneously affected internal anatomical structures. Different needle tissue area and MF were positively while TMF and CCF were negatively correlated with NSA. Interestingly, although both MA and TMA increased upon increasing NSA, MF showed a significantly negative correlation relationship with TMF. This may suggest a trade-off between photosynthetic tissue and supporting tissue when NSA increased and, seen from this study, this trade-off was mainly driven by NW. Our results add to the previous findings on the relationship between leaf size and investment to supporting tissues [9, 49, 50], suggesting that, in the studied species with narrow NL range, NW may also play a vital role in affecting needle anatomical traits and the resulting trade-off between photosynthetic tissue and supporting tissue may be related to surface exposure to incoming light. In addition, the correlation relationship among anatomical traits indicate that various needle inner structures may be developmentally interrelated [7, 44].

Biologists seek to distill organismal variation down to a manageable number of key functional traits [22, 51], within which leaf traits may fall along a ‘fast–slow’ continuum [52]. In other words, variations in needle traits, for conifers, may impose strong constraints on physiological functions and may furtherly affect plant survival and development. From a functional perspective, needle anatomical characteristics may be more directly related to physiological processes [26, 27]. For example, larger mesophyll tissue or vascular bundles may lead to higher photosynthetic capacity [14] or material transport capacity [53]. Additionally, LMA, one of the key leaf traits, has been demonstrated to negatively affect photosynthesis by reducing CO_2_ diffusion in many studies. These assumptions have been studied at both large and small scales [22–24], but seldom addressed in genus *Picea*, especially in species introduction trials when evaluating plant adaptability to new environments. In one of our previous studies, we found that net photosynthetic rate was positively correlated with some needle anatomical traits such as MA and CCA across 17 *Picea* species (including the five species in this study). However, the effects of needle internal anatomical structures as well as needle biochemical parameters on the differences of potential photosynthetic capacity among species remained unknown to a large extent [7]. Here, a more comprehensive study was conducted aiming at figuring all these relationships out. We found that the variation in the inherent photosynthetic capacity among the studied species were not related to the variation in LMA and needle anatomy (Table S1) but resulted mainly from the differences in g_s_, g_m_ and biochemical parameters including V_cmax_ and J_max_ in this study (Fig. 3). Moreover, the results of Grey correlative degree analysis (GRA) showed that g_m_, V_cmax_ and J_max_ contributed more to the interspecies variation in P_nmax_ (Fig. S1). g_s_, g_m_ and biochemical parameters including V_cmax_ and J_max_ were not correlated with needle anatomy and LMA, either (Table S1). Interestingly, we found that increased NSC (positively correlated with LMA, Fig. 2) was associated with decreased g_m_ among species, which has been seldom reported previously. NSC produced by photosynthesis is essential in plant functioning, being the substrates and energy sources for metabolic processes [54, 55]. Our results indicate that the accumulation of NSC may lower mesophyll CO_2_ diffusion across species, but this mechanism requires more research. In summary, two conclusions may be drawn from these results. Firstly, tissue-level anatomy is not sufficient to explain the variations in photosynthetic capacity among species. Although tissue-level anatomical traits are highly expected to be related to physiological functions as they are directly involved in physiological processes, among which photosynthetic capacity may be more related to cell-level anatomical traits such as mesophyll and chloroplast surface area exposed to intercellular air space [22, 56–58] and the contribution of different ultracellular and cellular components [22]. Secondly, for the studied species in genus *Picea*, robust leaf structure did not impose any constraints on g_m_ as well as photosynthetic capacity. Increased LMA is expected to be related to lower g_m_ [24, 28], but there are also exceptions; for example, both LMA and g_m_ would increase upon increased mesophyll cell surface area. This result was consistent with the findings in seven Mediterranean oaks species reported by Peguero-Pina et al. [22] and the findings in wild and domesticated cotton reported by Lei et al. [58], which may reflect the multiple determinants of LMA. Therefore, further study should be aimed at evaluating the relationship between needle finer-scale anatomy and LMA and, more importantly, better figuring out the coordination or trade-offs among needle functional traits and physiological processes underlying plant growth and adaptability in *Picea* breeding programs.

We found that increased g_s_, g_m_, V_cmax_ and J_max_ could result in higher P_nmax_. Additionally, iWUE scaled with both g_m_/g_s_ and V_cmax_/g_s_ (Fig. 3), suggesting that the two parameters can be considered as potential targets to improve iWUE [59, 60]. When considered under a large sample size including herbaceous, shrub and tree species, the two ratios were poorly correlated with each other and the absence of correlation indicated that improving each of them separately would improve iWUE [60]. However, g_m_/g_s_ was tightly correlated with V_cmax_/g_s_ in this study (Fig. 4), which implied that an enhanced iWUE required improving both g_m_/g_s_ and V_cmax_/g_s_ simultaneously. The discrepancies between this work and previous studies may be attributed to the limited samples involved in this study. Generally, increased g_s_ will simultaneously increase transpiration rate and decrease water use efficiency [24, 61]. Therefore, if g_s_ was tightly correlated with g_m_ and V_cmax_, it may be challenging to increase water-use efficiency without sacrificing photosynthetic rate. Although increasing g_m_ or V_cmax_ alone would result in increasing photosynthesis, and potentially increasing WUE, in practice, higher iWUE seems to be only achieved when there are no parallel changes in g_s_ [59]. In this study, g_s_ was weakly correlated with g_m_ and not correlated with V_cmax_ (Fig. S2, Fig. 3). In addition, no significant correlation relationship was detected between iWUE and P_nmax_ across species (Fig. S2). These results made it possible to identify species with both relatively high photosynthetic rate and water use efficiency—*P. likiangensis* showed the second highest P_nmax_ and the highest iWUE among the five studied species. For the other four species, they exhibited contrasting P_nmax_ but similar and low iWUE values (Table 2). Here comes the question—why don’t all species have high photosynthetic rate while maintaining high water-use efficiency? Muir et al. [24] proposed that other trade-offs in nature like water or N limitation can also have a negative effect on g_m_ or biochemical parameters, which would furtherly affect photosynthetic capacity. For example, a habitat with much water but little N may be beneficial to leaves with high stomatal density but low biochemical investment [61, 62] without altering the selection on overall leaf structure. These results suggest that the adjustments of water and N use efficiencies (PNUE and WUE) may also play an important role in determining the adaptability of plant species as the coordination or trade-offs along leaf spectrum do [24], even though no obvious coordination or trade-offs between needle structures and physiological processes were detected in this study (Table S1). P_nmax_, PNUE and WUE progressively increases along the phylogenetic tree of land plants [19], but when comparing different species or subjecting a species to e.g. water stress, both P_nmax_ and PNUE decrease concomitantly with increased WUE [63, 64]. This negative correlation relationship was also found in invasive vs. native species [65, 66] and transgenic Bt-cotton vs. its conventional peer [67]. Although PNUE was positively correlated with P_nmax_ in this study, it did not show any correlation with iWUE (Fig. S3). However, all studied species showed low PNUE but high iWUE, which is consistent with the results reported by Flexas and Carriquí [19], in which gymnosperms presented the largest WUE but relatively low PNUE through the phylogeny and they were not significantly correlated with each other.

Many studies have demonstrated that P_nmax_ increases with N_area_ [68], but this relationship is also dependent on species [69]. For example, an exception that P_nmax_ was inversely correlated with N_area_ in five southern pine species native to southeastern America was reported by Wang et al. [9]. In this study, we found that variations in P_nmax_ across species was not related to N_area_ (Table S1) but positively correlated with P_photo_ (Fig. 5). These results suggest that it is the fraction of N allocated to photosynthetic apparatus rather than needle total N content that is the key adaptive mechanism to maximize photosynthesis [70, 71]. In addition, both P_nmax_ and PNUE were significantly positively correlated with not only P_nmax_ but also P_L_, P_B_ and P_C_. This result is consistent with the findings reported in some common tress species in subtropical China [71, 72]. In this study, P_photo_, P_L_, P_B_ and P_C_ varied tremendously among species, with P_C_ being the largest in them for each species (Table 3), which has been well documented in previous studies (e.g., [33]). However, P_photo_ (3.23–18.41%) and P_C_ (1.64–11.96%) showed much lower values than those reported in previous studies; for example, P_photo_ and P_C_ of *Fagaceae* and *Leguminosae* tree species ranged between 13.86 and 27% and between 9.8 and 20%, respectively [73]. The differences between this work and previous studies may be attributed to species differences-- *Picea* species may invest more N to protective and storage chemicals [9] than other species.

In this study, the five species exhibited significant differences in growth traits at the experimental site. *P. glauca* exhibited the best growth performance, with the largest tree height, DBH and the number of first lateral branches among the five species at age 11. *P. likiangensis* and *P. koraiensis* also exhibited good growth performance while *P. crassifolia* and *P. mariana* showed poor growth (Table 4, Fig. 6). The considerable growth differences among the five species were largely determined by the variations in P_nmax_ and PNUE across species. In addition, the growth period for *P. glauca* was 95 days at the experimental site and was longer than that of the other four species [3], which may also be associated with the best growth of *P. glauca* among the five species. Interestingly, the two species introduced from North America, *P. glauca* and *P. mariana*, showed diametrically opposite growth characteristics. *P. glauca* and *P. mariana* have been introduced into China for more than 20 years. Chen et al. [74] first reported that the growth of *P. glauca* and *P. mariana* planted in northeast China could surpass the native species, *P. koraiensis*, at age 4, 5 and 6, but no differences were observed between the two species. Similar findings with this study that the growth of *P. glauca* was superior than *P. mariana* and *P. crassifolia* grown in Gansu province were also reported by An et al. [75]. *P. glauca* and *P. mariana* originated from North America, and most of their allocation ranges overlap with each other. However, *P. glauca* mostly grows in nutrient-rich soil, while *P. mariana* mostly grows in soil with relatively poor nutrition [76]. Therefore, the contrasting growth performance of the two species may be related to the long-term adaptation to the environments of their original habitats.

As a final note, we wish to point out that, our study was carried out in a common garden, so current variation in the measured traits among species might be a combination of genetic differences and the adaptive nature in response to both historical and current environmental conditions at the experimental site [77]. Additionally, photosynthetic parameters were estimated at the leaf-level, while the actual consequences of various structural designs on carbon gain and water use must ultimately be evaluated from shoots (the functional unit) to crowns and canopies [9], which requires further work.

## Conclusions

To the best of our knowledge, this study is a comprehensive comparison of needle structures, biochemical parameters, photosynthetic-related traits and plant growth among multiple *Picea* species and we found that several relationships between them can be described at the species level. To address the hypothesis posed in the Introduction, we draw four main conclusions: (1) changes in needle anatomical structures across the five studied species are affected by needle size, especially by NW; (2) compared with tissue-level anatomy and LMA, needle biochemical parameters play a more important role in determining the interspecific variation in photosynthetic capacity; (3) P_nmax_ and PNUE were affected by the fraction of N allocated to photosynthetic apparatus rather than needle total N content; (4) the five studied *Picea* species vary widely in growth traits, and this variation is substantially related to the differences in P_nmax_ and PNUE among the species. These insights provide a better understanding of the existing relationship between different biophysical structures in genus *Picea* and will be beneficial for the identification and selection of *Picea* species for a specific site condition.

## Materials and methods

### Study site and field experiment

In 2008, seeds of the five *Picea* species were sown in seedbeds and after cultivation for 3 years, the seedlings were planted with 1.5 m × 1.5 m spacing at the same site in a nursery at the Research Institute of Forestry of Xiaolong Mountain in Gansu Province, China (34°28′50″ N, 105°54′37″ E). Details on filed design, climate type at the experimental site, and geographical information as well as climate data for the 5 species have been reported elsewhere [3, 7].

### Needle morphology and anatomy

Needle sampling and the measurements of morphological and anatomical traits were conducted following the procedure reported by Wang et al. [7]. Needle morphological traits included needle length (NL, 0.01 mm), needle width at the middle of the needle (NW, 0.01 mm), needle thickness (NT, 0.01 mm) and stomatal density (SD, No./mm^2^). Anatomical traits included needle section area (NSA, 0.01mm^2^), epidermis tissue area and fraction (ETA, 0.01mm^2^; ETF, %), mesophyll area (MA, 0.001mm^2^; MF, %), and central cylinder area and fraction (CCA, 0.01mm^2^; MF, %). Total mechanical tissue area and fraction (TMA, 0.01mm^2^; TMF, %) were determined as the sum of area and fraction of epidermis tissue and central cylinder, respectively [46].

### Growth trait measurements

In July 2019, plant height, diameter at breast height (DBH) and the number of first lateral branches (at age 11) of the five species were determined. Additionally, tree height at age 6 and 9 was also integrated in this study for analysis.

### Needle gas exchange measurements

Gas exchange measurements were all conducted in late July 2019. Light-response curves of current-year-old needles on intact upper-crown branches were measured using Li-Cor 6400 gas-exchange system (with red/blue light source, and 6400-22 L conifer chamber; Li-Cor Biosciences, Lincoln, NE, USA). Four trees of each species were randomly selected, and one set of measurements were taken from each tree (*n* = 4). Photosynthetic response to photosynthetic photon flux density (PPFD) and C_i_ were determined on the same one healthy shoot. Under 400 μmol·mol^− 1^ of leaf chamber CO_2_ concentration, photosynthetic rates were measured at photon flux densities of 1800, 1600, 1200, 1000, 800, 600, 400, 200, 100, 75, 50, 25, 0 μmol·m^− 2^·s^− 1^ after each leaf sample was illuminated with a saturating level of PPFD for 10–30 min to achieve fully photosynthetic induction. Photosynthetic responses to variations in leaf internal CO_2_ concentration, i.e. *A-Ci* curves, were measured on the same shoot of each species where light-response curves were determined (*n* = 4). For each sample, a single curve over 16 chamber CO_2_ concentrations (400、300、200、100、60、400、400、600、800、1000、1200、1400、1600、2000、2300、2600 μmol·mol^− 1^) was measured. Maximum carboxylation rate (V_cmax_), and maximum electron transport rate (J_max_) were estimated from A–Ci curves using the plantecophys R package [78, 79]. In addition, the data of A-Ci curves were uploaded in the LeafWeb server (http://www.leafweb.org/) in order to have an automated analysis for mesophyll conductance (g_m_) [73, 80].

### Determination of LMA (leaf mass per unit area), chlorophyll and N content

Following gas exchange measurements, needles were carefully harvested and then pictured for needle area calculation using ImageJ software. Afterwards, needles were weighed and frozen in liquid N_2_. For leaf dry mass measurements, needles near the shoots for gas exchange measurements were collected, pictured and weighed as described above. After being oven-dried at 65 °C for 48 h and weighed, leaf mass per area (LMA, g m ^− 2^) was calculated as the ratio of needle dry mass(g) to needle area(m^2^).

Dried needle samples were ground into fine powder in a ball mill, needle N concentration (N_mass_, mg/g) was determined by a by high-temperature combustion using a LECO elemental analyzer (Leco Corporation, St-Joseph, Michigan). N_mass_ was then converted to a projected area basis (N_area_) using the LMA measurements for each sample. PNUE was determined as the ratio of P_nmax_ to N_area_.

Absolute chlorophyll concentration measurements were conducted using 95% (v/v) alcohol extracts of needle tissue and a Shimadzu visible-ultraviolet spectrophotometer (UV 2250, Fukuoka, Japan) following the procedure raised by Tang et al. [73].

### Calculation of nitrogen allocation in the photosynthetic apparatus

Nitrogen allocation fractions of each component in the photosynthetic apparatus were calculated according to Niinemets and Tenhunen [81].

$${P}_L=\frac{C_C}{C_B\times {N}_{mass}}\times 100\%$$

$${P}_B=\frac{J_{max}}{8.068\times {J}_{mc}\times {N}_{area}}\times 100\%$$

$${P}_C=\frac{V_{C\ \mathit{\max}}}{6.25\times {V}_{cr}\times {N}_{area}}\times 100\%$$

$${P}_{photo}={P}_L+{P}_B+{P}_C$$

$${P}_{non- photo}=1-{P}_{photo}$$

Where 6.25 is the N conversion coefficient into Rubisco (g Rubisco g^− 1^leaf N) [82] and 8.068 is the N binding coefficient for cytochrome f [83]. C_C_ is the chlorophyll concentration (mmol g ^− 1^). V_cr_ is the specific activity of Rubisco (20.78 umol CO_2_ g^− 1^ Rubisco s^− 1^) at 25 °C, C_B_ is the ratio of chlorophyll to organic N in light harvesting components (2.15 mmol g^− 1^) at 25 °C and J_mc_ is the potential rate of photosynthetic electron transport (155.65 μmol electrons μmol ^− 1^ Cyt f s ^− 1^) at 25 °C [81]. P_C_, P_B_, and P_L_ are the fraction of leaf nitrogen allocated to Rubisco, bioenergetics, and the light-harvesting components (%), respectively. P_photo_ indicated the leaf N allocated to the photosynthetic apparatus (P_Photo_) while P_non-photo_ indicated the leaf nitrogen allocated to non-photosynthetic apparatus.

### Determination of non-structural carbohydrate content (NSC)

The concentrations of soluble sugar and starch were determined using the anthrone method as described by Li et al. [84]. NSC was calculated as the sum of the concentration of soluble sugar plus the starch concentration.

### Statistical analysis

The normality of all data and the homogeneity of variance were tested prior to statistical analysis. An ANOVA to evaluate the species effect on growth traits of each year was carried out using the following model:

1 $${y}_{ij k}=\mu +{S}_i+{B}_j+{SB}_{ij}+{e}_{ij k}$$

where y_ijk_ is the observed value of species *i* in block *j*; *μ* is the mean value of the species; *S*_*i*_ is the random effect of species *i* = 1,2, …,5; *B*_*j*_ is the fixed effect of block *j* = 1, 2, 3, 4; *SB*_*ij*_ is the random effect of the interaction of species *i* and block *j*. *e*_*ijk*_ is the random error.

The effect of species on needle gas exchange and needle structures were tested using one-way analysis of variance (ANOVA):

2 $${y}_{ij}=\mu +{S}_i+{e}_{ij}$$

where y_ij_ is the observed value of sample needle; *μ* is the mean value of the species; *S*_*i*_ is the effect of species (random); and *e*_*ij*_ is the random error.

Duncan’s multiple range test was used to identify the differences in those different traits among tree species. In addition, means and standard deviations for each trait were estimated.

ANOVA and Duncan’s multiple range test were performed by SAS 9.4 (SAS Institute Inc., Raleigh, NC). Pearson correlation analysis and linear regression analysis were conducted by R (R, The University of Auckland, Auckland, New Zealand). In all analysis, significance in statistics was evaluated by *P* < 0.05.

## Supplementary Information

**Additional file 1.**

## Data Availability

The data supporting the findings of this study were all shown in the figures and tables and are available from the corresponding author upon reasonable request.

## References

[1] Farjon A. World checklist and bibliography of conifers. Royal Botanic Gardens, Kew, London. 2001

[2] Ren XW (1997). Dendrology.

[3] Ouyang FQ, Ma JW, Wang JC, Kong LS, Zhang HG, Tigabu M (2020). *Picea* species from humid continental and temperate marine climates perform better in monsoonal areas of middle latitudes of China. J For Res.

[4] Zhang SZ, Li XW, Yang ZS. Study on Early Selection of Picea koraiensis. J Northeast For Univ. 1982;3:36-44 (in Chinese)

[5] Xia Y, Zhang JW, Wang JH, Tao XM, Wang MH, Liu J (2014). Early valuation of eighteen provenances from five species of spruce. J Northeast Forest Univ.

[6] Ling JJ, Xiao Y, Hu JW, Wang FD, Ouyang FQ, Wang JH (2020). Genotype by environment interaction analysis of growth of *Picea koraiensis* families at different sites using BLUP-GGE. New Forest.

[7] Wang JC, Ma JW, OuYang FQ, Wang JH, Song L, Kong LS (2021). Instrinsic relationship among needle morphology, anatomy, gas exchanges and tree growth across 17 *Picea* species. New Forest.

[8] Holst M (1963). Growth of Norway spruce (*Picea abies* (L.) karst.) provenances in eastern North America.

[9] Wang N, Palmroth S, Maier CA, Domec J, Oren R (2019). Anatomical changes with needle length are correlated with leaf structural and physiological traits across five *Pinus* species: pine needle anatomy and physiology. Plant Cell Environ.

[10] Jasin’ska AK, Boratyn’ska K, Sobierajska K, Romo A, Ok T, Kharat MBD (2013). Relationships among *Cedrus libani*, *C. brevifolia* and *C. atlantica* has revealed by the morphological and anatomical needle characters. Plant Syst Evol.

[11] Higuchi H, Sakuratani T, Utsunomiya N (1999). Photosynthesis, leaf morphology, and shoot growth as affected by temperatures in cherimoya (*Annona cherimola* mill.) trees. Sci Hortic Amsterdam.

[12] Niinemets U, Lukjanova A, Turnbull MH, Sparrow AD (2007). Plasticity in mesophyll volume fraction modulates light-acclimation in needle photosynthesis in two pines. Tree Physiol.

[13] Xu CY, Salih A, Ghannoum O, Tissue DT (2012). Leaf structural characteristics are less important than leaf chemical properties in determining the response of leaf mass per area and photosynthesis of *Eucalyptus saligna* to industrial-age changes in [CO_2_] and temperature. J Exp Bot.

[14] Lin JX, Ceulemans MEJ (2001). Stomatal density and needle anatomy of scots pine (*Pinus sylvestris*) are affected by elevated CO_2_. New Phytol.

[15] Weng C, Jackson ST (2000). Species differentiation of north American spruce (*Picea*) based on morphological and anatomical characteristics of needles. Can J Bot.

[16] Guet J, Fabbrini F, Fichot R, Sabbati M, Bastien C (2015). Genetic variation for leaf morphology, leaf structure and leaf carbon isotope discrimination in European populations of black poplar ( *Populus nigra* L.). Tree Physiol.

[17] Niinemets Ü (2015). Is there a species spectrum within the world-wide leaf economics spectrum? Major variations in leaf functional traits in the Mediterranean sclerophyll *Quercus ilex*. New Phytol.

[18] Gifford RM, Evans L (1981). Photosynthesis, carbon partitioning, and yield. Annu Rev Plant Physiol.

[19] Flexas J, Carriquí M (2020). Photosynthesis and photosynthetic efficiencies along the terrestrial plant's phylogeny: lessons for improving crop photosynthesis. Plant J.

[20] Wright IJ, Reich PB, Westoby M, Ackerly DD, Baruch Z, Bongers F (2004). The worldwide leaf economics spectrum. Nature.

[21] Wright IJ, Reich PB, Cornelissen JHC, Falster DS, Groom PK, Hikosaka K (2005). Modulation of leaf economic traits and trait relationships by climate. Glob Ecol Biogeogr.

[22] Peguero-Pina JJ, Sisó S, Flexas J, Galmés J, García-Nogales A, Niinemets Ü (2017). Cell-level anatomical characteristics explain high mesophyll conductance and photosynthetic capacity in sclerophyllous Mediterranean oaks. New Phytol.

[23] Flexas J, Ribas-Carbó M, Diaz-Espejo A, Galmés J, Medrano H (2008). Mesophyll conductance to CO_2_: current knowledge and future prospects. Plant Cell Environ.

[24] Muir CD, Conesa MÀ, Roldán EJ, Molins A, Galmés J (2017). Weak coordination between leaf structure and function among closely related tomato species. New Phytol.

[25] Niinemets Ü, Díaz-Espejo A, Flexas J, Galmés J, Warren CR (2009). Role of mesophyll diffusion conductance in constraining photosynthetic productivity in the field. J Exp Bot.

[26] Evans JR (1999). Leaf anatomy enables more equal access to light and CO_2_ between chloroplasts. New Phytol.

[27] Meng JX, Chen XY, Huang YJ, Wang LM, Xing FQ, Li Y (2019). Environmental contribution to needle variation among natural populations of *Pinus tabuliformis*. J Forestry Res.

[28] Niinemets Ü, Wright IJ, Evans JR (2009). Leaf mesophyll diffusion conductance in 35 Australian sclerophylls covering a broad range of foliage structural and physiological variation. J Exp Bot.

[29] Terashima I, Hanba YT, Tholen D, Niinemets Ü (2011). Leaf functional anatomy in relation to photosynthesis. Plant Physiol.

[30] Tomás M, Flexas J, Copolovici L, Galmés J, Hallik L, Medrano H (2013). Importance of leaf anatomy in determining mesophyll diffusion conductance to CO_2_ across species: quantitative limitations and scaling up by models. J Exp Bot.

[31] Sharwood RE, Crous KY, Whitney SM, Ellsworth DS, Ghannoum O (2017). Linking photosynthesis and leaf N allocation under future elevated CO_2_ and climate warming in *Eucalyptus globulus*. J Exp Bot.

[32] LeBauer DS, Treseder KK (2008). Nitrogen limitation of net primary productivity in terrestrial ecosystems is globally distributed. Ecology.

[33] Hou WF, Tränkner M, Lu JW, Huang SY, Ren T, Cong RH (2019). Interactive effects of nitrogen and potassium on photosynthesis and photosynthetic nitrogen allocation of rice leaves. BMC Plant Biol.

[34] Evans IR (1989). Photosynthesis and nitrogen relationships in leaves of C3 plants. Oecologia.

[35] Evans JR, Seemann JR, Briggs WR (1989). The allocation of nitrogen in the photosynthetic apparatus: costs, consequences and control. Photosynthesis.

[36] Makinom A, Osmondm B (1991). Solubilization of ribulose-1 5-bisphosphate carboxylase from the membrane fraction of pea leaves. Photosynth Res.

[37] Poorter H, Evans JR (1998). Photosynthetic nitrogen-use efficiency of species that differ inherently in specific leaf area. Oecologia.

[38] Walcroft AS, Whitehead D, Silvester WB, Kelliher FM (1997). The response of photosynthetic model parameters to temperature and nitrogen concentration in *Pinus radiata* D. Don Plant Cell Environ.

[39] Hikosaka K, Osone Y (2009). A paradox of leaf-trait convergence: why is leaf nitrogen concentration higher in species with higher photosynthetic capacity?. J Plant Res.

[40] Hikosaka K (2004). Interspecific difference in the photosynthesis-nitrogen relationship: patterns, physiological causes, and ecological importance. J Plant Res.

[41] Robinson DE, Wagner RG, Bell FW, Swanton CJ (2001). Photosynthesis, nitrogen-use efficiency, and water-use efficiency of jack pine seedlings in competition with four boreal forest plant species. Can J For Res.

[42] Feng YL, Lei YB, Wang RF, Callaway RM, Valiente-Banuet A, Inderjit N (2009). Evolutionary trade-offs for nitrogen allocation to photosynthesis versus cell walls in an invasive plant. Proc Natl Acad Sci U S A.

[43] Hikosaka K (2010). Mechanisms underlying interspecific variation in photosynthetic capacity across wild plant species. Plant Biotechnol NAR.

[44] Huang Y, Mao J, Chen Z, Meng J, Xu Y, Duan A, Li Y (2016). Genetic structure of needle morphological and anatomical traits of *Pinus yunnanensis*. J For Res.

[45] Pickup M, Westoby M, Basden A (2005). Dry mass costs of deploying leaf area in relation to leaf size. Funct Ecol.

[46] Kuusk V, Niinemets Ü, Valladares F (2018). A major trade-off between structural and photosynthetic investments operative across plant and needle ages in three Mediterranean pines. Tree Physiol.

[47] Raupach MR, Thom AS (1981). Turbulence in and above plant canopies. Annu Rev Fluid Mech.

[48] Vogel S (1989). Drag and reconfiguration of broad leaves in high winds. J Exp Bot.

[49] Niinemets Ü, Portsmuth A, Tobias M (2006). Leaf size modifies support biomass distribution among stems, petioles and mid-ribs in temperate plants. New Phytol.

[50] Niinemets Ü, Sack L (2006). Structural determinants of leaf-harvesting capacity and photosynthetic potentials. In Progress in botany (ed. W. Beyschlag).

[51] Pérez-Harguindeguy N, Díaz S, Garnier E, Lavorel S, Poorter H, Jaureguiberry P (2013). New handbook for standardized measurement of plant functional traits worldwide. Aust J Botany.

[52] Mason CM, Donovan LA (2015). Evolution of the leaf economics spectrum in herbs: evidence from environmental divergences in leaf physiology across *Helianthus* (Asteraceae). Evolution.

[53] Ghimire B, Lee C, Heo K (2013). Leaf anatomy and its implications for phylogenetic relationships in Taxaceae sl. J Plant Res.

[54] Hartmann H, Trumbore S (2016). Understanding the roles of nonstructural carbohydrates in forest trees-from what we can measure to what we want to know. New Phytol.

[55] Hartmann H, Adams HD, Hammond WM, Hoch G, Landhäusser SM, Wiley E (2018). Identifying differences in carbohydrate dynamics of seedlings and mature trees to improve carbon allocation in models for trees and forests. Environ Exp Bot.

[56] Peguero-Pina JJ, Flexas J, Galmés J, Niinemets Ü, Sancho-Knapik D, Barredo G (2012). Leaf anatomical properties in relation to differences in mesophyll conductance to CO_2_ and photosynthesis in two related Mediterranean Abies species [J]. Plant Cell Environ.

[57] Lu Z, Ren T, Li J, Hu WS, Zhang JL, Yan JY (2020). Nutrition-mediated cell and tissue-level anatomy triggers the covariation of leaf photosynthesis and leaf mass per area. J Exp Bot.

[58] Lei ZY, Liu F, Wright IJ, Carriquí M, Niinemets Ü, Han JM, et al. Comparisons of photosynthetic and anatomical traits between wild and domesticated cotton. J Exp Bot. 2021:erab293. 10.1093/jxb/erab293 34153103

[59] Flexas J, Niinemets Ü, Galle A, Barbour MM, Centrito M, DiazEspejo A (2013). Diffusional conductances to CO_2_ as a target for increasing photosynthesis and photosynthetic water-use efficiency. Photosynth Res.

[60] Gago J, Douthe C, Florez-Sarasa I, Escalona JM, Galmés J, Fernie AR (2014). Opportunities for improving leaf water use efficiency under climate change conditions. Plant Sci.

[61] Wright IJ, Reich PB, Westoby M (2003). Least-cost input mixtures of water and nitrogen for photosynthesis. Am Nat.

[62] Bloom AJ, Chapin FS, Mooney HA (1985). Resource limitation in plants - an economic analogy. Annu Rev Ecol Syst.

[63] van den Boogard R, Kostadinova S, Veneklaas E, Lambers H (1995). Association of water use efficiency and nitrogen use efficiency with photosynthetic characteristics of two wheat cultivars. J Exp Bot.

[64] Heckathorn SA, De Lucia EH, Zielinski R (1997). The contribution of drought-related decreases in foliar nitrogen concentration to decreases in photosynthetic capacity during and after drought in prairie grasses. Physiol Plant.

[65] Durand ZE, Goldstein G (2001). Photosynthesis, photoinhibition, and nitrogen use efficiency in native and invasive tree ferns in Hawaii. Oecologia.

[66] Funk JL, Vitousek PM (2007). Resource-use efficiency and plant invasion in low-resource systems. Nature.

[67] Guo R, Sun S, Liu B (2016). Difference in leaf water use efficiency/photosynthetic nitrogen use efficiency of Bt-cotton and its conventional peer. Sci Rep.

[68] Evans JR, Clarke VC (2019). The nitrogen cost of photosynthesis. J Exp Bot.

[69] Kattge J, Díaz S, Lavorel S, Prentice IC, Leadley P, Bönisch G (2011). TRY - a global database of plant traits. Glob Chang Biol.

[70] Qin RM, Zheng YL, Valiente-Banuet A, Callaway RM, Barclay GF, Pereyra CS (2013). The evolution of increased competitive ability, innate competitive advantages, and novel biochemical weapons act in concert for a tropical invader. New Phytol.

[71] Tang JC, Sun BD, Cheng RM, Shi ZM, Luo D, Liu SR (2019). Effects of soil nitrogen (N) deficiency on photosynthetic N-use efficiency in N-fixing and non-N-fixing tree seedlings in subtropical China. Sci Rep.

[72] Tang JC, Sun BD, Cheng RM, Shi ZM, Luo D, Liu SR (2019). Seedling leaves allocate lower fractions of nitrogen to photosynthetic apparatus in nitrogen fixing trees than in non-nitrogen fixing trees in subtropical China. PLoS One.

[73] Tang JC, Cheng RM, Xu GX, Liu SR, Centritto M (2018). *Fagaceae* tree species allocate higher fraction of nitrogen to photosynthetic apparatus than *Leguminosae* in Jianfengling tropical montane rain forest, China. PLoS One.

[74] Chen XB, Zhang MH (1996). Preliminary report on introduction test of *Picea mariana* and *P. glauca*. Jilin forestry. Sci Technol.

[75] An SP, Xu N, Du YC, Wang LF, Ma JW, Wang JH (2018). Early evaluation of growth traits of *Picea* species and provenances. For Res.

[76] Power H, Schneider R, Berninger F (2014). Understanding changes in black (*Picea mariana*) and white spruce (*Picea glauca*) foliage biomass and leaf area characteristics. Trees.

[77] Jankowski A, Wyka TP, Żytkowiak R, Danusevičius D, Oleksyn J (2019). Does climate-related in situ variability of scots pine (*Pinus Sylvestris* L.) needles have a genetic basis? Evidence from common garden experiments. Tree Physiol.

[78] Duursma RA (2015). Plantecophys - an R package for analysing and modelling leaf gas exchange data. PLoS One.

[79] Xiong DL, Flexas J (2021). Leaf anatomical characteristics are less important than leaf biochemical properties in determining photosynthesis responses to nitrogen top-dressing. J Exp Bot.

[80] Gu LH, Pallardy SG, Tu K, Law BE, Wullschleger SD (2010). Reliable estimation of biochemical parameters from C3 leaf photosynthesis-intercellular carbon dioxide response curves. Plant Cell Environ.

[81] Niinemets Ü, Tenhunen JD (1997). A model separating leaf structural and physiological effects on carbon gain along light gradients for the shade-tolerant species *Acer saccharum*. Plant Cell Environ.

[82] Jordan DB, Ogren WL (1984). The CO_2_/O_2_ specifcity of ribulose 1,5-bisphosphate carboxylase/oxygenase: dependence on ribulose bisphosphate concentration, pH and temperature. Planta.

[83] Nolan WG, Smillie RM (1977). Temperature-induced changes in hill activity of chloroplasts isolated from chilling-sensitive and chilling-resistant plants. Plant Physiol.

[84] Li MH, Jiang Y, Wang A, Li XB, Zhu WZ, Yan CF (2018). Active summer carbon storage for winter persistence in trees at the cold alpine treeline. Tree Physiol.

