# Supplementary material for: Variation, coordination, and trade-offs between needle structures and photosynthetic-related traits across five Picea species: consequences on plant growth
Source: BMC Plant Biol. 2022 May 17;22:242. doi: 10.1186/s12870-022-03593-x (PMC9112436; doi:10.1186/s12870-022-03593-x)
Supplement: Supplementary file 1 — Additional file 1. [file 12870_2022_3593_MOESM1_ESM.docx]

Table S1 Pearson’s correlation coefficients for relationships between needle structures and photosynthetic capacity and biochemical parameters

|  | g_s_ | g_m_ | V_cmax_ | J_max_ | iWUE | P_nmax_ |
| --- | --- | --- | --- | --- | --- | --- |
| NL | 0.07 | 0.44 | 0.55* | 0.60* | 0.28 | 0.48* |
| NW | -0.33 | -0.06 | -0.02 | 0.02 | 0.12 | -0.32 |
| NT | -0.30 | -0.05 | 0.00 | 0.07 | 0.10 | -0.15 |
| SD | 0.43 | 0.43 | 0.45 | 0.43 | 0.15 | 0.66** |
| MA | -0.36 | 0.07 | 0.18 | 0.18 | 0.54* | -0.03 |
| CCA | -0.44 | -0.26 | -0.14 | -0.02 | -0.03 | -0.31 |
| TMA | -0.20 | 0.29 | 0.45 | 0.36 | 0.36 | 0.16 |
| MF | -0.29 | -0.17 | -0.21 | -0.10 | 0.34 | -0.18 |
| CF | -0.17 | -0.42 | -0.43 | -0.29 | -0.48* | -0.37 |
| TMF | 0.28 | 0.25 | 0.30 | 0.17 | -0.29 | 0.24 |
| LMA | -0.10 | -0.35 | -0.42 | -0.27 | -0.47* | -0.35 |
| N_area_ | -0.10 | 0.14 | 0.02 | -0.01 | 0.00 | -0.15 |
| NSC | -0.17 | -0.57* | -0.60** | -0.44 | -0.52* | -0.45 |

NL, needle length; NW, needle width ; NT, needle thickness; SD, stomatal density; NSA, needle section area; ETA and ETF, epidermis tissue area and fraction; MA and MF, mesophyll area and fraction; CCA and CCF, central cylinder area and fraction; TMA and TMF, total mechanical tissue area and fraction; LMA, leaf mass per area; N_area_, leaf N content per area; NSC, non-structural carbohydrate content; P_nmax_, area-based maximum net photosynthesis; g_s_, stomatal conductance; iWUE, intrinsic water use efficiency; g_m_, mesophyll conductance to CO_2_; V_cmax_ and J_max_, maximum carboxylation and maximum electron transport rate, respectively. *, statistical significance at *P* < 0.05; **, statistical significance at *P* < 0.01.


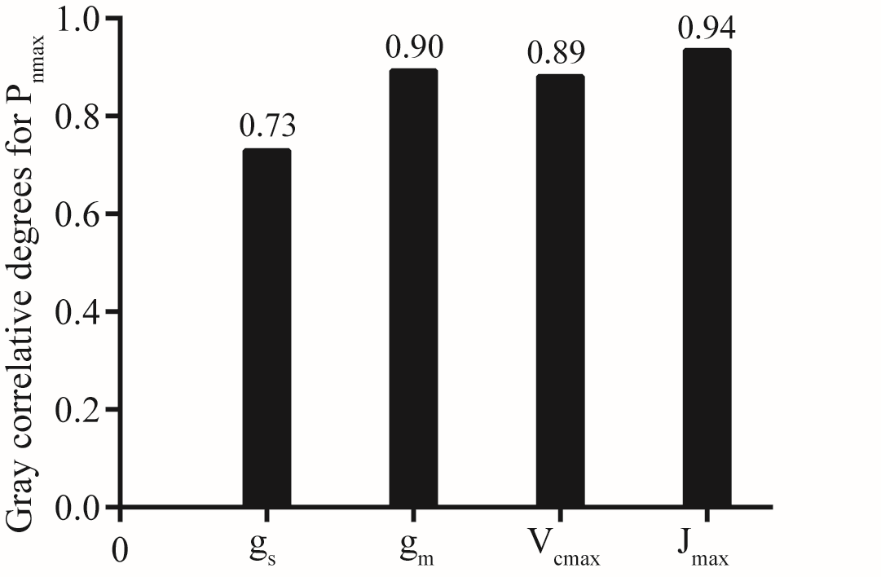


Figure S1 Grey correlative degree analysis of biochemical parameters for P_nmax_. P_nmax_, area-based maximum net photosynthesis; g_s_, stomatal conductance; g_m_, mesophyll conductance to CO_2_; V_cmax_ and J_max_, maximum carboxylation and maximum electron transport rate, respectively. Data are grey correlative degree, which indicate the contribution of different traits for the variation in P_nmax_.


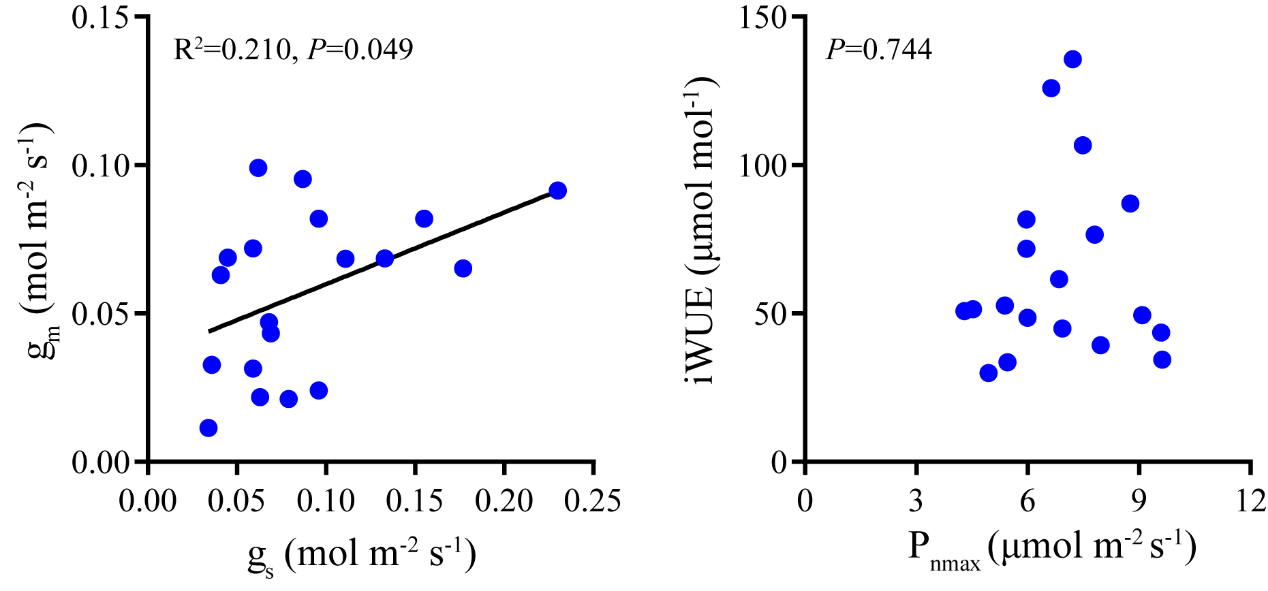


Figure S2 Relationships between g_s_ and g_m_ and between P_nmax_ and iWUE. g_s_, stomatal conductance; g_m_, mesophyll conductance to CO_2_; P_nmax_, area-based maximum net photosynthesis; iWUE, intrinsic water use efficiency. Points represent trait values for each individual of each species. Explained variance (R^2^) and *P* values are shown. Solid lines in each panel represents significant linear regression.


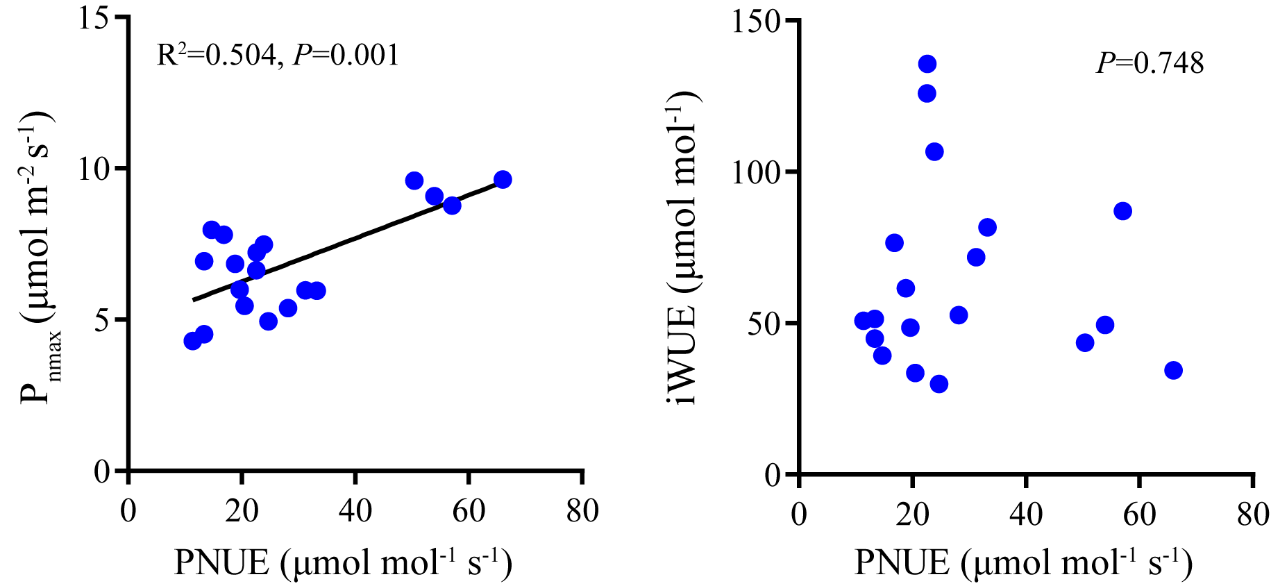


Figure S3 Relationships between PNUE and P_nmax_ as well as iWUE. P_nmax_, area-based maximum net photosynthesis; iWUE, intrinsic water use efficiency; PNUE, photosynthetic nitrogen use efficiency. Points represent trait values for each individual of each species. Explained variance (R^2^) and *P* values are shown. Solid lines in each panel represents significant linear regression.
